# Tobamovirus 3′-Terminal Gene Overlap May be a Mechanism for within-Host Fitness Improvement

**DOI:** 10.3389/fmicb.2017.00851

**Published:** 2017-05-11

**Authors:** Yuri L. Dorokhov, Ekaterina V. Sheshukova, Tatiana V. Komarova

**Affiliations:** ^1^N.I. Vavilov Institute of General Genetics, Russian Academy of ScienceMoscow, Russia; ^2^A.N. Belozersky Institute of Physico-Chemical Biology, Lomonosov Moscow State UniversityMoscow, Russia

**Keywords:** overlapping genes, plant virus, virus genome, evolution, tobamovirus, movement protein, coat protein

## Abstract

Overlapping genes (OGs) are a universal phenomenon in all kingdoms, and viruses display a high content of OGs combined with a high rate of evolution. It is believed that the mechanism of gene overlap is based on overprinting of an existing gene. OGs help virus genes compress a maximum amount of information into short sequences, conferring viral proteins with novel features and thereby increasing their within-host fitness. Analysis of tobamovirus 3′-terminal genes reveals at least two modes of OG organization and mechanisms of interaction with the host. Originally isolated from *Solanaceae* species, viruses (referred to as *Solanaceae*-infecting) such as tobacco mosaic virus do not show 3′-terminal overlap between movement protein (MP) and coat protein (CP) genes but do contain open reading frame 6 (ORF6), which overlaps with both genes. Conversely, tobamoviruses, originally isolated from *Brassicaceae* species (referred to as *Brassicaceae*-infecting) and also able to infect *Solanaceae* plants, have no ORF6 but are characterized by overlapping MP and CP genes. Our analysis showed that the MP/CP overlap of *Brassicaceae*-infecting tobamoviruses results in the following: (i) genome compression and strengthening of subgenomic promoters; (ii) CP gene early expression directly from genomic and dicistronic MP subgenomic mRNA using an internal ribosome entry site (IRES) and a stable hairpin structure in the overlapping region; (iii) loss of ORF6, which influences the symptomatology of *Solanaceae*-infecting tobamoviruses; and (iv) acquisition of an IRES polypurine-rich region encoding an MP nuclear localization signal. We believe that MP/CP gene overlap may constitute a mechanism for host range expansion and virus adjustment to *Brassicaceae* plants.

## Introduction

Overlapping genes (OGs) are a universal phenomenon in all kingdoms. OGs originate via various mechanisms, including the use of alternative start codons, ribosomal read through, and frameshift mutations (Krakauer, 2000). Viruses, particularly RNA viruses, possess a high content of OGs in addition to a high mutation rate and a high rate of evolution (Belshaw et al., 2008; Simon-Loriere et al., 2013; Geoghegan et al., 2017).

There are several explanations for the abundance of overlapping genes in RNA viruses. The appearance of OGs (i) helps viruses to compress a maximum amount of information into short sequences (Belshaw et al., 2008) and increase their fitness in various hosts (Krakauer, 2000), (ii) constitutes a mechanism of gene expression regulation via translational coupling of functionally related polypeptides (Belshaw et al., 2007), and (iii) represents an effective mechanism for novel gene generation by introducing a new open reading frame (ORF) over an existing one (Pavesi et al., 2013; Simon-Loriere et al., 2013; Saha et al., 2016).

Tobacco mosaic virus (TMV), the first virus to be discovered, along with other members of the tobamovirus group may provide information about the possible roles of OGs. It is believed that tobamoviruses have coevolved with their hosts (co-divergence) since formation of the asterid, rosid, and caryophyllid plant lineages approximately 112.9 million years ago (Gibbs et al., 2015). A second mechanism of primordial tobamovirus evolution is more rapid, as when there is a sudden appearance of a new host plant, which presumably occurs due to human agricultural activity (host switching) (Gibbs et al., 2010). If we take into account Vavilov's ideas about the centers of origin of cultivated plants (Vavilov and Dorofeev, 1992), it is difficult to imagine a different mechanism because the centers of *Solanaceae* and *Brassicaceae* plant origin are on different continents. The current host-virus pair reflects the within-host fitness of tobamovirus to the host plant.

Here, we attempt to resolve the putative role of tobamovirus 3′-terminal gene overlap in within-host fitness.

## 3′-terminal gene overlap and tobamovirus classification

The genome of viruses originally isolated from *Solanaceae*- species (referred to as *Solanaceae*-infecting) (such as the U1 strain) contains six ORFs and encodes six proteins, four of which are a 126-kDa protein (ORF2) and its read-through derivative of 183-kDa (ORF1), a 30-kDa protein (ORF4) or movement protein (MP), and a coat protein (CP) (ORF5) (Cooper, 2014). The fifth, a 54-kDa protein (ORF3), has an undefined role, if any. The sixth, a 4.8-kDa protein encoded by ORF6, which overlaps ORF4 and ORF5, influences symptomatology but is not found in all tobamovirus species (Canto et al., 2004). The 130-kDa and 180-kDa proteins are translated directly from the genomic RNA, whereas MP and CP are synthesized from their respective subgenomic RNAs that are 5′-capped and 3′-coterminal with the genomic RNA. TMV MP mRNA is bicistronic; as in the genomic RNA, the MP and CP genes do not overlap and are separated by a 3-nt spacer.

**Figure 1:**
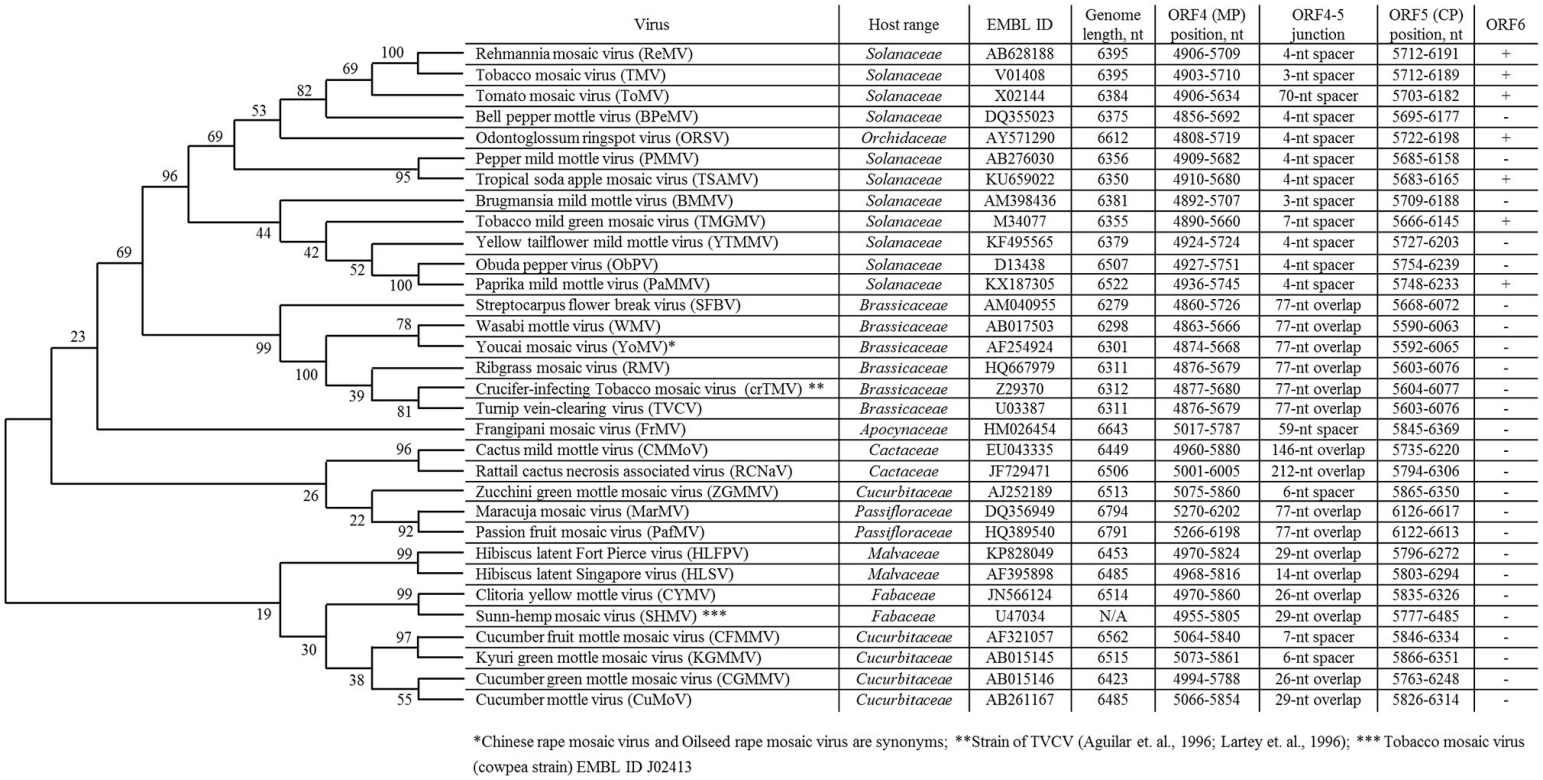
**List of overlapping tobamovirus MP/CP genes and tobamovirus molecular phylogenetic analysis based on amino acid sequences of CP proteins using the maximum likelihood method using the JTT matrix-based model (Jones et al., 1992)**. Evolutionary analyses were conducted in MEGA7 (Kumar et al., 2016).

Currently, the genus *Tobamovirus* consists of 35 species (ICTV, virus taxonomy: 2015 release, http://www.ictvdb.org/virusTaxonomy.asp), and TMV is the type species. The natural host ranges for different members of the genus *Tobamovirus* include *Solanaceae, Brassicaceae, Cucurbitaceae, Malvaceae, Cactaceae, Passifloraceae, Fabaceae, Apocynaceae, Cannabaceae*, and *Orchidaceae* (Figure 1). Initially, tobamoviruses were classified into two subgroups (Fukuda et al., 1981) with different genomic locations for their origin of virion assembly (OA). The OA is located within the MP gene in subgroup 1 members, including *Solanaceae*-infecting tobamoviruses; an exception is odontoglossum ringspot virus, which infects orchids. In subgroup 2 tobamoviruses, such as those infecting *Cucurbitaceae* and *Fabaceae*, the OA is contained within the CP gene.

In 1994, we first obtained the complete nucleotide sequence of the crucifer-infecting tobamovirus (crTMV) isolated from a *Brassicaceae* plant, garlic mustard (*Alliaria petiolata*) (Dorokhov et al., 1994), which later came to be regarded by ICTV as a strain of turnip vein-clearing virus (TVCV) (Aguilar et al., 1996; Lartey et al., 1996). The genome organization of crTMV is different from that of other tobamoviruses, as the MP and CP ORFs overlap by 77 nt (Table 1). Once the complete nucleotide sequences of the other two viruses originally isolated from *Brassicaceae* species (referred to as *Brassicaceae*-infecting) became available (Aguilar et al., 1996; Lartey et al., 1996), a third subgroup of the genus was proposed (Aguilar et al., 1996; Lartey et al., 1996). The OAs of viruses from subgroup 3 are also located in the MP ORF; however, the MP and CP ORFs overlap by 77 nt, and members of this subgroup are able to infect both *Solanaceae* and *Brassicaceae* species. Isolation of new tobamoviruses, such as frangipani mosaic virus (Lim et al., 2010), maracuja mosaic virus (Song et al., 2006), passion fruit mosaic virus (Song and Ryu, 2011), cactus mild mottle virus (Min et al., 2006) and rattail cactus necrosis-associated virus (Kim et al., 2012), along with the complete sequencing of their genomes, showed that MP/CP overlap is not unique to *Brassicaceae-*infecting tobamoviruses but is also present in *Apocynaceae*-, *Passifloraceae*-, and *Cactaceae*-infecting viruses (Figure 1).

**Table 1:** **Sequence analysis of tobamoviruses with 3-terminal gene overlap**.

**Tobamovirus**	**EMBL ID and genome length (nt)**	**Host range**	**MP gene polypurine-rich region**			**MP-CP gene overlapping region**		
			**Nucleotide sequence**	**Encoded amino acid sequence**	**Distance from CP gene (nt)**	**Length (nt)**	Sequence (nt)^***^	**^#^RNA folding, stem-loop structure ΔG (kcal/mol)**
Turnip vein-clearing virus (TVCV)	U03387, 6311	*Brassicaceae*	GGAAAAGGAAAAAGAAGGTTGAAGAAAAGGGAT	209-RKRKKKVEERD-219	70	77	**ATG**TCTTACAACATTACAAACCCGAATCAGTACCAGTACTTCGCAGCGGTGTGGGCAGAGCCCATACCAATGCTTAA	−16.3
Crucifer-infecting Tobacco mosaic virus (CrTMV)^*^	Z29370, 6312		AAAAGAAGGAAAAAGAAGGTTGAAGAAAAGGGT	209-KRRKKKVEEKG-219	70	77	**ATG**TCTTACAACATTACAAACCCGAATCAGTACCAGTATTTCGCAGCGGTGTGGGCAGAGCCCATTCCGATGCTTAA	−13.7
Ribgrass mosaic virus (RMV)	HQ667979, 6311		AGGAAAAAGAAAAGGAAGATAGGAGGAAGTGAAG	206-RKKKKKIGGRD-216	69	77	**ATG**TCTTACAACATCACGAGCTCGAATCAGTACCAGTATTTCGCAGCGGTGTGGGCAGAGCCCACACCAATGCTTAA	−22.1
Wasabi mottle virus (WMV)	AB017503, 6298		AGGAAAAAGAAAAAGAAGATTGGAGGAAGGGAT	209-RKKKKKIGGRD-219	71	77	**ATG**TCATACAACATCACGAACTCGAATCAGTACCAGTTTTTCGCAGCGGTATGGGCGGAGCCCATAGCAATGCTTAA	−13.4
Youcai mosaic virus (YoMV)^**^	AF254924, 6301		AGAAAGAAAAAGAAAAAGATTGGAGGAAAGGAT	206-RKKKKKIGGKD-216	74	77	**ATG**TCTTACAACATCACGAGCTCGAATCAGTACCAGTATTTCGCAGCGATGTGGGCAGAGCCCACAGCGATGCTTAA	−17.9
Passion fruit mosaic virus (PafMV)	HQ389540, 6791	*Passifloraceae*	AAGGAGAAAGAAAGG	258-RRKK-261	71	77	**ATG**CCTTATCAACCGGTTTCTCTACAGACACTTCCATGGCTGTCGGCCAACTGGGCCGATTACAAGACACTTCT**TAG**	−13.2
Maracuja mosaic virus (MarMV)	DQ356949, 6794		GAGGAAAAAGAAAGG	258-RKKK-261	71	77	**ATG**CCTTACCAACCAATAACGATCAAGCAGTTACCATGGGTGTCCGCCAACTGGGCGGATTACCAAACACTGGT**TAA**	−18.7
Cactus mild mottle virus (CMMoV)	EU043335, 6449	*Cactaceae*	GAAAAGAAAAGATTGAGAAAAGG	229-EKKRLRKG-236	60	146	**ATG**GCGGGTTCTTACACCAACGTGAAGCCGAACACATTTGTGTACCGGACTCAGTCGTGGGTCGAACCGGAAAAATTACTCAATTACTTAACCCAGGCGCAACTTCTCATCTTTCAGACGCAGCAAGCTCGGACACAACTTGC**TAA**	−21.5
Rattail cactus necrosis-associated virus (RCNaV)	JF729471, 6506		AAGGGTCGTAGAAGTAGAGGTAAA	233-KGRRSRGK-240	73	212	**ATG**CCTTACATCAACGTACAACCGAAAGACTTCGTTTACCTGACCAGGTCATGGGTAGACCCCCAGCGTCTGATACAGTTTCTGAGCGATATGCAGACTCAGGCTTTCCAAGCACAGCAGTCTCGCACACAACTTCTGAACGAACTGTCGACGATGGTGGTCTACGGTCCGACCAAATCGGACCGTTTCCCTATAGATACGTACCTTAT**TAA**	−11.8
Streptocarpus flower break virus (SFBV)	AM040955, 6279	*Gesneriaceae*	NA	NA	NA	77	**ATG**GGCGGATCCAATTCAATTGATGAATCTTTGTTCAGCGTCGCTGAGTCAGATCTTTCAGACTCAGGCTGCGCG**TGA**	−18.6

* *Strain of TVCV (Lartey et al., 1995; Aguilar et al., 1996)*.

** *Synonymous with Chinese rape mosaic virus or oilseed rape mosaic virus*.

*** *The CP start codon and MP stop-codon are in bold; the hairpin-forming region is underlined*.

# *Hairpin prediction was performed by the “UNAFold” service (http://unafold.rna.albany.edu)*.

The MP/CP overlap of *Brassicaceae*-infecting tobamovirus is characterized by a +1 frameshift, which is the most frequent for viral gene overlap (Belshaw et al., 2007), yet the mechanisms supporting the evolutionary stability of MP/CP OGs remains unclear. It is known that for a random missense mutation in an overlapping region to remain in the population, this mutation must be beneficial for one of the genes and at least neutral for the other (Brandes and Linial, 2016). The origin and function of the 126/183-kDa OGs confirm a general idea of the origin of eukaryotic and viral OGs (Belshaw et al., 2007) regarding their association with regulation of gene expression via the translational coupling of functionally related polypeptides. Nonetheless, the role of MP/CP OGs functional coupling is unknown.

Unlike the 126- and 183- kDa proteins involved in RNA viral replication functions, MP and CP perform diverse functions. MP is a nonstructural protein, the properties of which are revealed by its interaction with cellular factors associated with the cell-to-cell transport of macromolecules. In contrast, CP is a structural protein, and its amino acid sequence is determined by the mechanism of virion assembly and formation. It can be assumed that joint evolution of primordial virus MP and CP genes led to their overlap, which allowed the virus to utilize *Brassicaceae* as a host in the evolutionary host-switching process.

Below we define a possible benefit for acquisition of MP/CP overlap by *Brassicaceae*-infecting tobamoviruses.

## Influence of MP/CP overlap on CP mRNA synthesis

Confirmation of the dominant role of gene overlap in genome compression can be found in the size of the genome of tobamoviruses that infect both *Brassicaceae* and *Solanaceae* and have a 77-nt MP/CP overlap, making their genomic RNA shorter (6298-6312 nt) in comparison with those infecting only *Solanaceae* (6350-6522 nt) (Figure 1) (Belshaw et al., 2008). However, the size of genomes containing overlapping MP/CP genes in *Passifloraceae*-infecting tobamoviruses (PafMV and MarMV), with genome lengths close to 6800 nt, does not agree with this assumption (Figure 1). One possible explanation for the appearance of MP/CP gene overlap in tobamovirus evolution is the necessity of strengthening the activity of subgenomic promoters directing the synthesis of a dicistronic and monocistronic mRNA encoding MP and CP, respectively. This view is primarily supported by the results of Dawson et al. (Culver et al., 1993), who observed the earlier and higher expression of MP when the gene was closer to the 3′-nontranslated region (NTR), which contains three pseudoknots followed by a tRNA-like structure. CP gene proximity to pseudoknots also determines gene expression (Dawson, 2014).

The 3′-NTR of *Brassicaceae*-infecting tobamoviruses is ~30 nt longer than the 3′-NTR of those infecting only *Solanaceae*; however, as we have shown, crTMV genomic RNA contains six pseudoknots and one additional pseudoknot at the 3′ end of the CP gene (Dorokhov et al., 1994). We can assume that the maximum proximity of the CP gene to pseudoknots promotes CP RNA synthesis from a subgenomic promoter.

Thus, the MP/CP gene overlap of *Brassicaceae*-infecting tobamoviruses should be evaluated in terms of not only genome compression but also the contribution of strengthening subgenomic promoters as the distance to 3′-NTR pseudoknots is decreased.

## Influence of MP/CP overlap on CP mRNA translation

TMV CP, in addition to its primary functions of virion assembly and systemic virus movement, is involved in the formation of virus replication complexes (VRCs) in the early stage of infection (Asurmendi et al., 2004). Moreover, CP supports early synthesis of MP, which facilitates the rapid movement of viral genetic material (Bendahmane et al., 2002).

We can assume that early synthesis of CP creates favorable conditions for the virus effective cell-to-cell movement and long-distance spreading. In general, tobamoviruses are characterized by the capacity for rapid and early synthesis of viral proteins, ensuring rapid movement of viral genetic material through the plant and accumulation of viral particles in quantities exceeding the yields of other plant viruses. Apparently, rapid synthesis of proteins and rapid movement from cell to cell allows tobamoviruses to overcome the development of host protective reactions. A benefit with regard to expanding the plant host range is conferred on the virus, which is able to synthesize CP not only from subgenomic mRNA but also from genomic or MP directing dicistronic I_2_-RNA. *Brassicaceae*-infecting tobamoviruses appear to have such an advantage, as they are capable of early synthesis of CP through internal translation initiation.

Indeed, internal initiation of translation has been shown for MP gene expression in both TMV U1 and crTMV (Zvereva et al., 2004). In addition, we also revealed that the sequence of crTMV genomic RNA upstream of the CP gene contains a 148-nt internal ribosome entry site (IRES_148,CP_^CR^) (Ivanov et al., 1997), which can mediate translation initiation in yeast, plant and animal systems *in vitro* and *in vivo* from both dicistronic constructs and constructs with blocked 5′-end-dependent translation (Dorokhov et al., 2002). Sequence analysis of IRES_CP,148_^CR^ revealed a stem-loop hairpin structure surrounded on both sides by a polypurine-rich region (PPR) with a long 33-nt PPR33 before and a short 11-nt PPR11 after the hairpin. Moreover, PPR33 is sufficient for IRES_CP,148_^CR^ activity, and a simplified version of such a sequence, consisting of only 16 (GAAA) repeats, also enables high cross-kingdom efficiency of cap-independent translation initiation in plant and mammalian cells (Dorokhov et al., 2002). It should be noted that the corresponding 148-nt region in the TMV U1 RNA exhibits no homology with IRES_CP,148_^CR^ and is not able to direct internal initiation of translation (Ivanov et al., 1997). Thus, unlike TMV U1, crTMV CP appears to be translated both from subgenomic mRNA by a cap-dependent mechanism and from dicistronic I_2_-RNA or genomic RNA via internal translation initiation.

To examine the possibility of CP synthesis in a plant cell directly from crTMV genomic RNA, we created a binary vector containing the crTMV infectious copy; in this system, the promoter controlling the synthesis of CP subgenomic mRNA was inactivated via introduction of nucleotide substitutions while IRES_CP,148_^CR^ remained intact (Dorokhov et al., 2006). Agroinjection experiments allowed us to establish the possibility of CP synthesis from genomic RNA. Moreover, we estimated that the contribution of IRES_CP,148_^CR^ to the total synthesis of CP during viral infection was at least 3%.

Although this experimental setup leaves some doubts about the IRES's ability to be active under natural conditions when the subgenomic promoter is functional, we hypothesized that internal translation initiation is important for the synthesis of CP, providing early formation of the VRC and enhanced systemic movement of the virus (Dorokhov et al., 2006).

Here, we have analyzed *Brassicaceae*-infecting tobamoviruses and other tobamoviruses with overlapping MP/CP genes to identify a common crTMV nucleotide sequence or signature for CP gene expression via an internal initiation translation mechanism. Table 1 shows that all examined tobamoviruses with overlapping MP/CP genes are characterized by two features that can contribute to internal translation initiation of the CP ORF. The first feature, previously noted for crTMV, is the PPR of the MP gene, located at a distance of 60–70 nt from the CP start codon. PPR lengths varies among viruses but is completely absent only in streptocarpus flower break virus. The second feature is the existence of a stable stem-loop hairpin structure at the end of the overlapping region, directly upstream of the MP gene termination codon (Table 1). It can be assumed that such a hairpin structure assists in ribosome recognition of the CP initiation codon during translation of the genomic RNA.

An important consequence of the presence of the PPR in the MP gene of *Brassicaceae*-infecting tobamoviruses is the emergence of an arginine and lysine-rich amino acid stretch (Table 1), which functions as a nuclear localization signal and enables entry of TVCV MP into the nucleus (Levy et al., 2013). This observation led the authors to reasonably presume a nuclear phase of TVCV MP biogenesis, one function of which is blocking host defense. It should be noted that the appearance of overlapping MP/CP genes in *Brassicaceae*-infecting tobamovirus evolution is accompanied by loss of nested and overlapping ORF6, which is typical of viruses infecting only *Solanaceae* (Figure 1). ORF6 functions as a pathogenicity factor (Canto et al., 2004), affecting the nuclear apparatus of infected cells (Gushchin et al., 2013; Erokhina et al., 2017). Thus, tobamoviruses may invoke at least two different strategies for influencing the host cell nucleus: (i) via ORF6 overlap with MP and CP genes, in tobamoviruses originally isolated from *Solanaceae* species, and (ii) by acquiring a new property of MP to infect *Brassicaceae*.

## Concluding remarks

Current host-virus pairs reflect the tobamoviruses within-host fitness together with optimal combination of tobamoviruses and their host plants. Therefore, the typical tobamovirus U1 strain is adapted to *Solanaceae* plants, and *Brassicaceae-*infecting viruses are adapted to the *Brassicaceae* plants from which they are isolated. An evolutionarily acquired ability to infect *Brassicaceae* plants is a manifestation of a common-to-all-tobamovirus strategy of genome expression characterized by rapid and early synthesis of viral proteins encoding 3′-terminal genes. Acquisition of MP/CP gene overlap leads to early and enhanced synthesis of CP, and loss of ORF6 by *Brassicaceae*-infecting tobamoviruses is somehow compensated for by acquisition of the ability to affect nuclear function by MP. MP/CP gene coupling and the evolutionary stability of the overlap could be explained by the benefit to both ORFs and is accompanied by overprinting of the C-terminal sequence of MP, which is not essential for the movement function.

Although our analysis is limited to *Solanaceae*- and *Brassicaceae*-infecting tobamoviruses, which are relatively well studied, 3′-terminal OGs are also observed in other viruses infecting other plant families. It can be assumed that their study may in the future reveal other mechanisms of tobamovirus within-host fitness.

## Author contributions

YD designed the sequence analysis; ES performed the bioinformatics analysis; YD and TK wrote the manuscript with input from all authors who reviewed the final paper.

## Funding

This work was supported by the Russian Science Foundation (project No. 16-14-00002).

### Conflict of interest statement

The authors declare that the research was conducted in the absence of any commercial or financial relationships that could be construed as a potential conflict of interest.
